# Supplementary material for: Label-free 3D molecular imaging of living tissues using Raman spectral projection tomography
Source: Nat Commun. 2024 Sep 9;15:7717. doi: 10.1038/s41467-024-51616-y (PMC11384735; doi:10.1038/s41467-024-51616-y)
Supplement: Supplementary file 1 — Supplementary Information [file 41467_2024_51616_MOESM1_ESM.pdf]

Supplementary information

Label-free 3-D molecular imaging of living tissues using Raman Spectral Projection Tomography

Elzbieta Stepula[1&], Anders R. Walther[2&], Magnus Jensen[1], Dev Mehrotra[3], Mu H. Yuan[1], Simon V. Pedersen[4], Vishal Kumar[1], Eileen Gentleman[1,5], Michael B. Albro[3], Martin A. B. Hedegaard[2,*], and Mads S. Bergholt[1,*]

[1]*Centre for Craniofacial & Regenerative Biology, King's College London, SE1 9RT London, United Kingdom*

[2]*SDU Chemical Engineering, Faculty of Engineering, University of Southern Denmark, Campusvej 55, 5230 Odense, Denmark*

[3]*Department of Mechanical Engineering, Boston University, Boston, Massachusetts, USA*

[4]*SDU Biotechnology, Faculty of Engineering, University of Southern Denmark, Campusvej 55, 5230 Odense, Denmark*

[5]*Department of Biomedical Sciences, University of Lausanne, 1005 Lausanne, Switzerland*

[&] *These authors contributed equally*

* *Corresponding authors*: mads.bergholt@kcl.ac.uk, marhe@igt.sdu.dk

Supplementary Methods

Benchmarking of the Raman spectral projection tomography instrument

The FOV was determined by the configuration of the imaging lenses, the linear fiber array length, as well as the width of the line-shaped laser beam. The imaging resolution depends on the magnification and ultimately the linear detection fiber array (50 µm fibers). The system was benchmarked using a FOV of 9 mm x 9 mm. The voxel size can be calculating by ratio of the FOV to the number of detectors (n=43). The magnification was calculated by dividing the size of the image on the CCD by the actual size of the object.[50, 51]

The contrast ratio of RSPT as a function along the optical axis was measured using a set of Raman active resolution targets that were 3D printed. The targets were designed with line patterns of varying sizes (1000 µm, 500 µm, and 400 µm) and positioned at a 45-degree angle to the optical axis. Projections were captured for each target, with an integration time of 30 seconds per projection. The contrast ratio was derived from the intensity variations between the resin signal and background.

The Modulation Transfer Function (MTF) of the optical system was measured using a projection of a cylindrical sample of resin. We opted to assess the MTF on a projected data since there was no computational bias introduced by applying the inverse radon for the reconstruction. We averaged the Edge Spread Function (ESF) from projections of a cylindrical phantom of resin. From this, the Line Spread Function (LSF) was derived by differentiating the ESF. The MTF was subsequently computed by evaluating the Fourier Transform of the LSF and determining its magnitude. The MTF was normalized by its peak value and then graphed against spatial frequency, providing an insight into the spatial resolution and overall image quality. A 5th order polynomial fitting was employed to represent the MTF, as showcased in Figure 2D, where the MTF was charted versus the spatial frequency (cycles per mm).

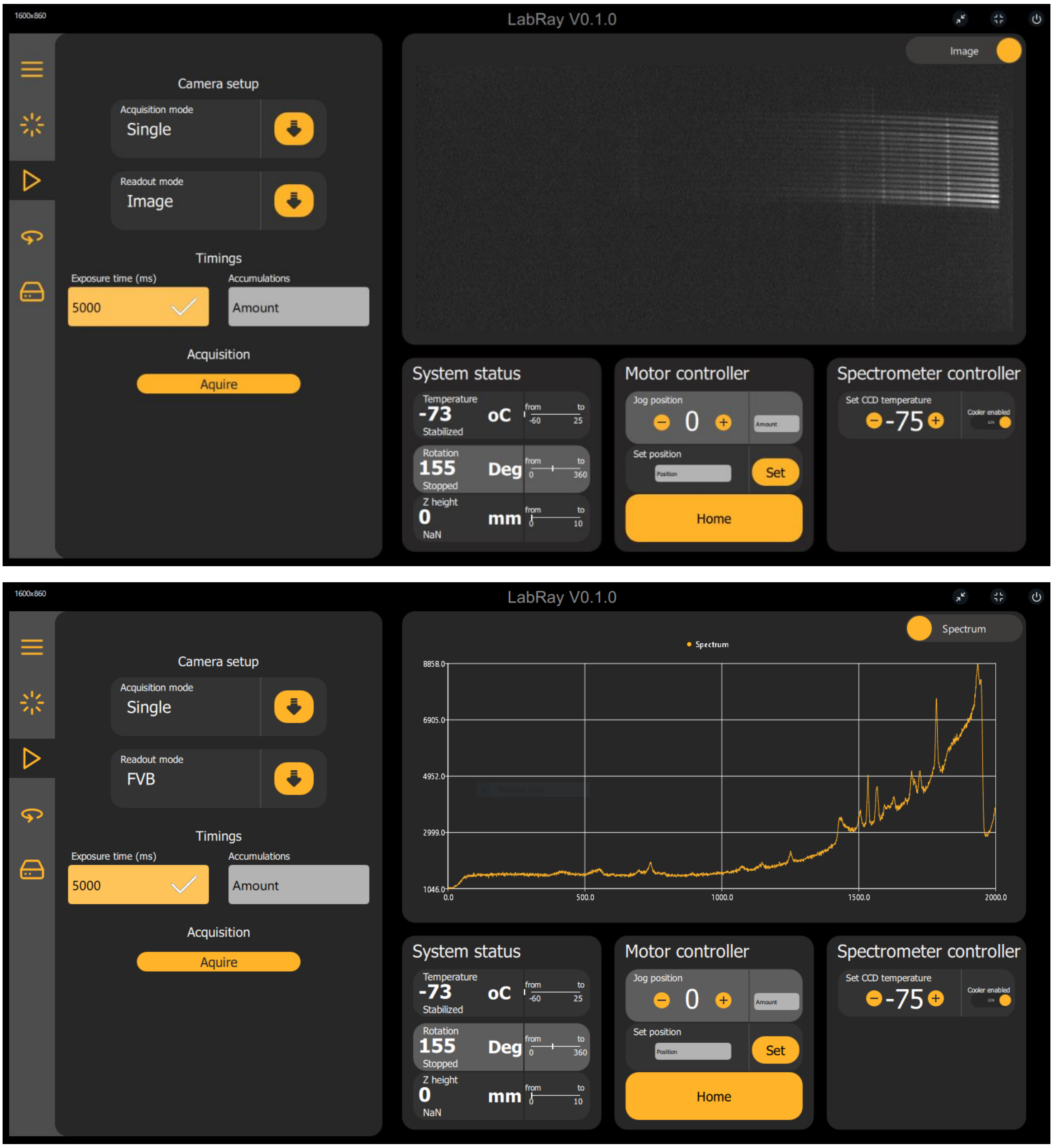

Figure S1 The Graphical User Interface (GUI) of the RSPT software, developed in Python, enables automated rotation and linear translation of the stage, facilitating the capture of a comprehensive tomographic dataset. Users can adjust parameters such as z-step height, rotational resolution, slice count, and integration time within the interface. Additionally, the software calculates and displays the total duration of the measurement in real-time. Users can monitor and verify the ongoing measurement through a live display of the average spectrum and the unprocessed CCD image.

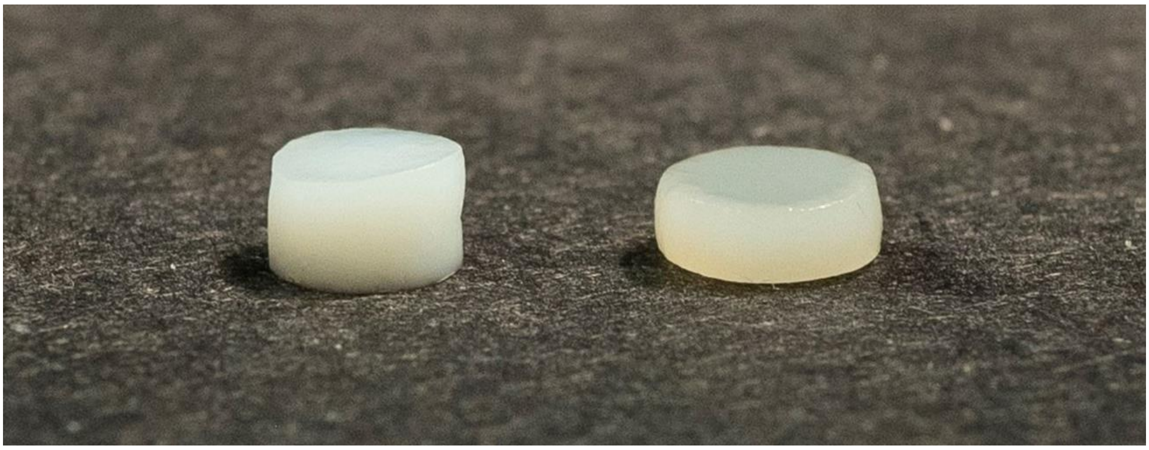

Figure S2 Comparison between native cartilage and tissue-engineered cartilage. The sample on the left side shows articular cartilage explants obtained from the medial and lateral condyles of 3-6-month-old bovine calves. On the right side, the sample exhibits tissue-engineered cartilage after 42 days of growth.

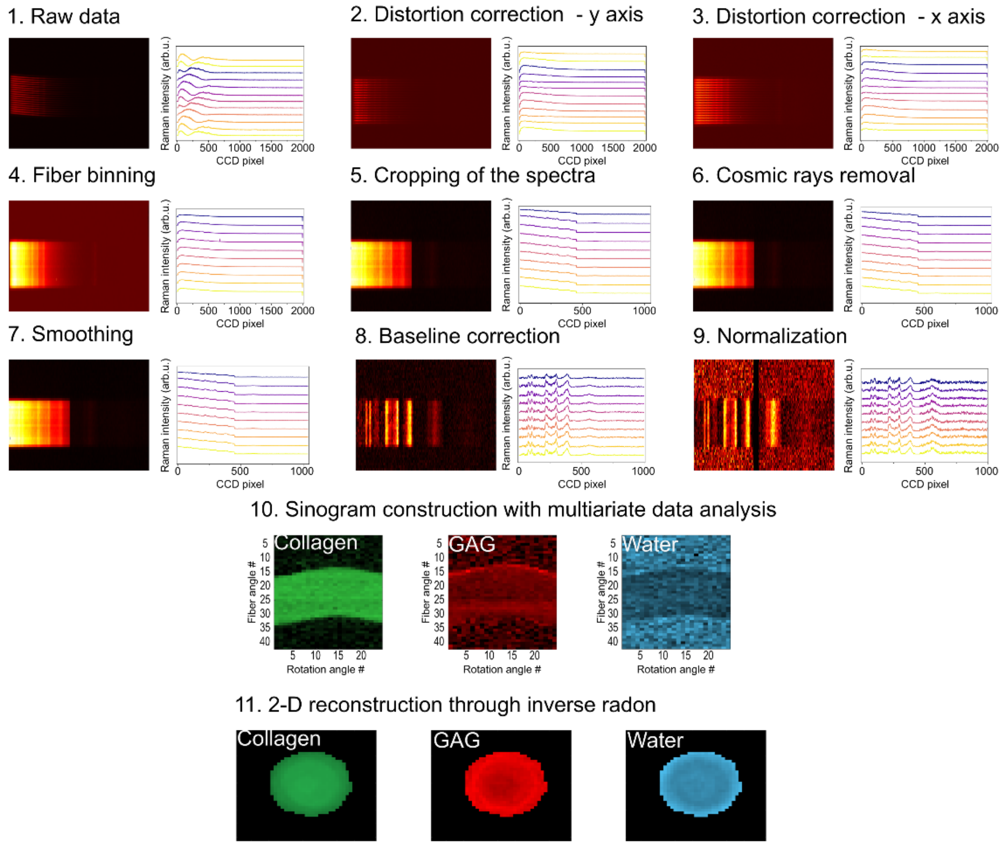

Figure S3 The RSPT reconstruction steps for molecular image reconstruction showcase CCD images and corresponding Raman spectra: (1) Raw data: The CCD image of the cartilage sample is presented along with 10 representative raw spectra from the sample region. (2) Distortion correction – y-axis: The processed CCD image is shown after polynomial function-based distortion correction. (3) Distortion correction – x-axis: Spectra are wavelength corrected using prior measurements from a standard resin block. (4) Fiber binning: Summation over every 5 pixels rows is performed, resulting in 43 Raman spectra. (5) Cropping to fingerprint and high wavenumber region: Spectra are confined to the fingerprint region (800 cm^{-1} to 1800 cm^{-1} and 2800 cm^{-1} to 3600 cm^{-1}). (6) Cosmic ray removal: The CCD image and spectra are shown post-removal of cosmic ray interference with a threshold. 7) Smoothing: Spectra are subjected to Savitzky-Golay smoothing. 8) Baseline correction: Raman spectra are shown post-baseline correction via 3rd order polynomial fitting (fingerprint) and 1st order (high-wavenumber). (9) Normalization: The CCD image is displayed alongside vector normalized spectra. (10) Sinogram construction: After abundance estimation using non-negativity constrained least squares, constructed sinograms represent glycosaminoglycan (GAG), collagen, water, and background. (11) 2-D reconstructions: Back-projected 2-D reconstructions are derived from the sinograms, and the reconstructions are shown after removing the reconstruction background with a threshold.

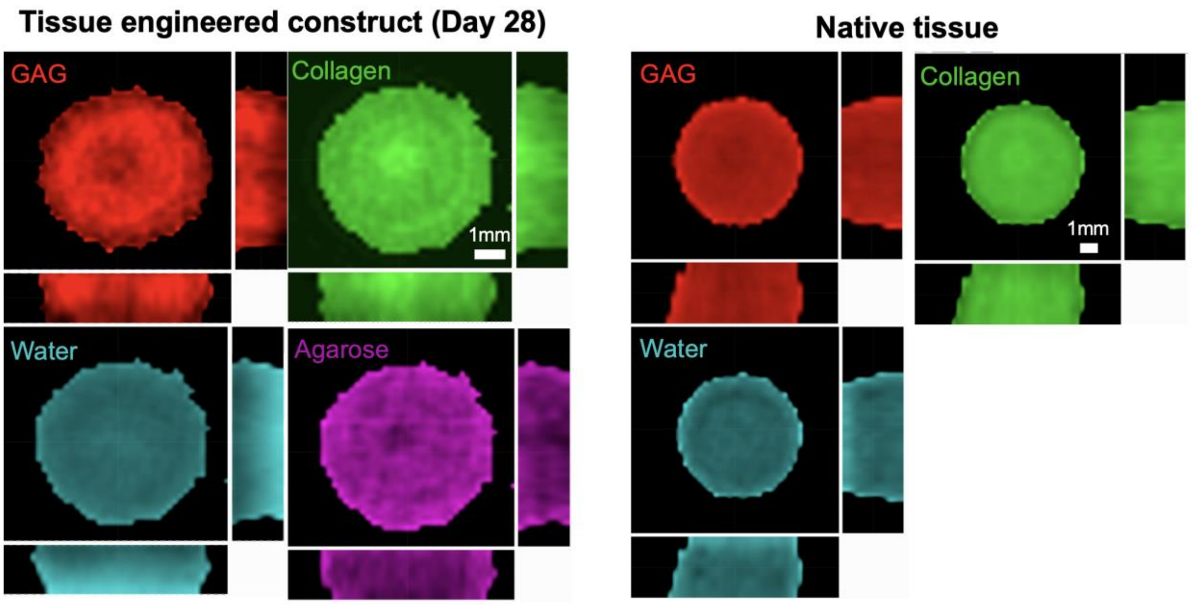

Figure S4 Two-dimensional slice visualizations of 3-D reconstructions from tissue engineered construct from day 28 (left side) and native tissue (right side) depicting the distributions of collagen, glycosaminoglycan (GAG), water and in the case of TE agarose. Central panel: XY orientation; lower panel: YZ orientation; right panel: XZ orientation.

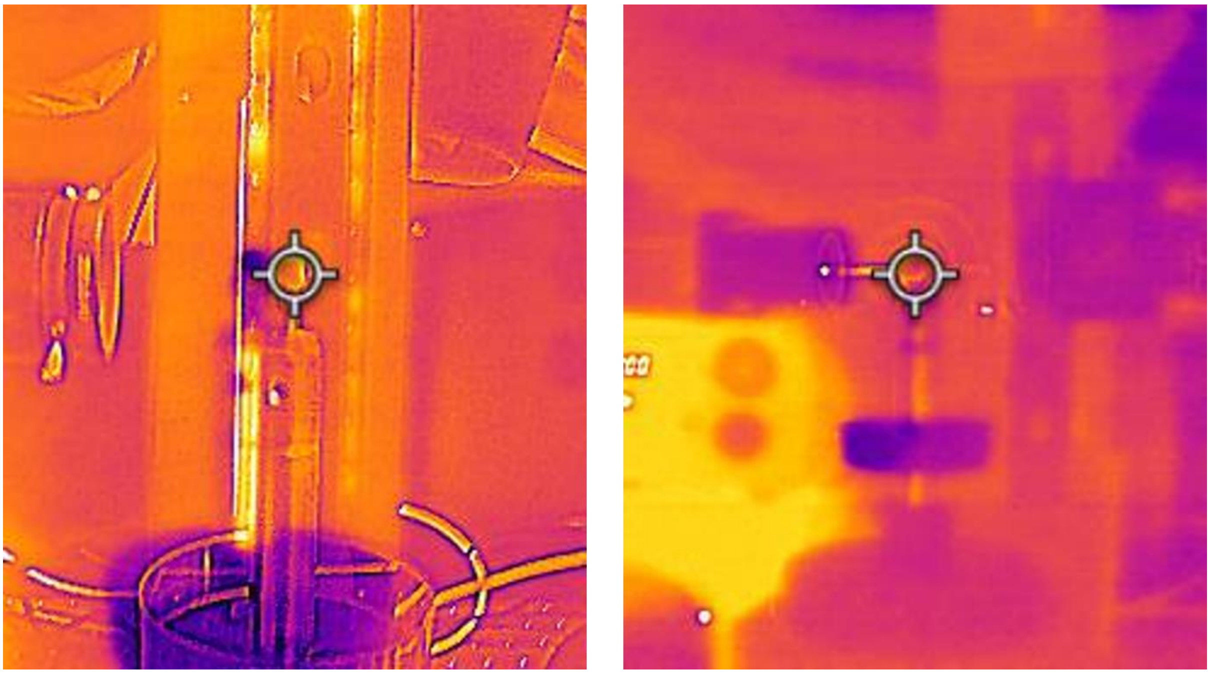

Figure S5 Thermal imaging using a FLIR camera was conducted on a tissue-engineered construct during an RSPT measurement. The image indicates no significant temperature increase in the construct compared to the surrounding area during laser exposure.
