# Supplementary material for: Label-free 3D molecular imaging of living tissues using Raman spectral projection tomography
Source: Nat Commun. 2024 Sep 9;15:7717. doi: 10.1038/s41467-024-51616-y (PMC11384735; doi:10.1038/s41467-024-51616-y)
Supplement: Supplementary file 3 — Description of Additional Supplementary Files [file 41467_2024_51616_MOESM3_ESM.pdf]

Description of Additional Supplementary Files

Supplementary Movie 1: 3D rotational tomography video of the cylindrical resin phantom sample: full rotation view

Supplementary Movie 2: 3D rotational tomography video of the cuboid resin phantom sample: full rotation view

Supplementary Movie 3: 3D rotational tomography video of the prism resin phantom sample: full rotation view

Supplementary Movie 4: 3D rotational tomography video of the two-component sample: a hollow resin cylinder encasing a PTEG prism: full rotation view

Supplementary Movie 5: 3D rotational tomography video of collagen in native cartilage: full rotation view

Supplementary Movie 6: 3D rotational tomography video of GAG in native cartilage: full rotation view

Supplementary Movie 7: 3D rotational tomography video of water in native cartilage: full rotation view
